# Lessons Learned from Longer Acting Reversible Contraception Applied to Longer Acting HIV Prevention Technologies

**DOI:** 10.1007/s11904-021-00571-0

**Published:** 2021-09-15

**Authors:** Rachel Logan, Dominika Seidman

**Affiliations:** 1 Tampa, USA; 2 Zuckerberg San Francisco General Hospital, 1001 Potrero Ave Ward 6D, San Francisco, CA 94110 USA

**Keywords:** Family planning, PrEP, LARC, Reproductive justice, Health equity, Sexual health

## Abstract

**Purpose of Review:**

This review describes lessons learned from longer acting contraception and employs a reproductive justice lens to inform expansion of emerging HIV prevention technologies.

**Recent Findings:**

Reproductive justice is a framework that advocates for the promotion of universal sexual and reproductive freedoms, particularly among historically marginalized communities. This framework takes a holistic view of individuals and sees the interconnections between sexual health, reproductive health, and overall health. Employing a sexual and reproductive justice perspective is essential to understanding and helping to mitigate the role intersecting structural, sexual, and reproductive oppressions, including those demonstrated through promotion of longer acting contraception, and can critically inform rollout of future prevention technologies, such as longer acting HIV pre-exposure prophylaxis.

**Summary:**

This review highlights the need for researchers, clinicians, and policymakers to apply lessons learned from contraception and specifically focuses on principles of reproductive justice to offer expanding HIV prevention options.

## Introduction

As HIV prevention technologies expanded over the past decade, researchers, clinicians, and advocates have frequently drawn parallels between contraception and HIV pre-exposure prophylaxis (PrEP) [1, 2]. While the connection is not surprising—clinical messaging around oral PrEP and oral contraceptives beg the comparison—preventing HIV and preventing pregnancy are clearly different [2, 3]. Nevertheless, in the context of currently available prevention technologies, public health efforts to prevent unwanted pregnancies and prevent HIV ultimately focus on cisgender women’s sexual health. Women’s bodies have been politicized for centuries [4] and continue to be so. In order to effectively offer HIV prevention technologies, we must acknowledge the historical and ongoing political context that informs individuals’ past and current experiences of sexual and reproductive health prevention services [5••].

In this paper, we offer a brief review of the expansion of contraceptive options, focusing on long-acting reversible methods (LARC). While LARC methods were heralded as a panacea for pregnancy prevention, their rollout demonstrates how easily an exclusive focus on method effectiveness provided opportunities for discrimination and coercion and silenced individuals’ preferences and goals. We then highlight the reproductive justice movement [6], founded by a group of Black women in the United States (U.S.) in the 1990s, as a way to reconceptualize prevention technologies beyond their effectiveness and refocus public health priorities on people’s health and well-being. Reproductive justice has always incorporated sexual health, though its inclusion is often muffled in the context of highly politicized debate over reproduction.

While many groups have faced and continue to experience reproductive oppressions, including transgender people and other gender diverse groups, we focus on cisgender women in this discussion as they were targeted for pregnancy prevention interventions vis-à-vis LARC while being frequently excluded from early HIV prevention technology development. Here, we apply principles of reproductive justice to the development of longer acting HIV prevention technologies and multipurpose technologies to avoid some of the harmful missteps that have occurred in the history of contraceptive technologies. While we use historical evidence that is focused on cisgender women, principles of reproductive justice may be applied to all people regardless of gender identity. In fact, given persistently high rates of HIV in transgender people worldwide, it may be particularly important to avoid some of the historical missteps and harms described here. Nevertheless, transgender and gender diverse people have experienced unique historical contexts and intersecting oppressions; a comprehensive discussion of lessons learned applied to PrEP provision for transgender and gender diverse people is beyond the scope of this review.

## Lessons Learned from LARC

LARC methods are highly efficacious in preventing pregnancy. Unlike other contraceptive methods where regular user application or dosing is required, LARC methods have the same theoretical effectiveness as their actual effectiveness. For this reason, LARC methods were seen as superior to other methods because they are highly effective and reversible. Professional clinical organizations [7–9] also recommended LARC methods as “first-line” contraception and trained contraceptive care providers to counsel using a tiered-effectiveness approach [10••]. In this counseling strategy, the effectiveness of a method, and therefore LARC, is prioritized above any other method characteristic.

LARC implementation highlighted the need to address interpersonal and systemic reproductive coercion. Broadly defined, reproductive coercion is the act of any individual, group, system, or policy that seeks to unduly influence, promote, or restrict options for someone to exercise their right to bodily and reproductive autonomy [11, 12]. Reasons for reproductive coercion are steeped in eugenics and stratified reproduction—determining who had the right to birth and parent children [4]. These beliefs often targeted groups with minoritized and stigmatized identities, such as unmarried people; poor people; youth; Black, Indigenous, People of Color (BIPOC); and other historically underserved and excluded groups, such as queer, trans, and non-binary people, sex workers, and those with mental and physical disabilities.

In this sociopolitical context, reproductive coercion was made manifest when state and local governments in the U.S. made LARC use compulsory for incarcerated or formerly incarcerated people and those who received public assistance or social services [4]. Other examples include the forcible insertion of LARC methods, abortion, and sterilization of people in China under the One-Child policy [13]. Less blatant, though likely more prevalent demonstrations include providers limiting options for patients by exclusively educating on and offering LARC, or providers refusing to remove LARC for those who wished to discontinue use [14–16]. Currently, similar groups who have been the “targets” of population-level public health interventions for pregnancy prevention are also those targeted for the prevention of sexually transmitted infections (STIs) and HIV. This focus on population-level outcomes can overshadow support of individual reproductive agency and autonomy [10••]. Ongoing challenges in contraceptive education and counseling remain to ensure people who want these methods have access to the full range of options without coercion [16, 17]; HIV prevention implementation risks the same challenges if an exclusive focus on method effectiveness and method use is employed.

## An Introduction to Reproductive Justice

In 1994, a group of Black women in the U.S. developed the reproductive justice framework in response to the reproductive oppression Black women were experiencing and their exclusion from reproductive and abortion rights movements. The underpinnings of reproductive justice are rooted in a global human rights–based approach [18] to sexual and reproductive health and serve as a declaration that Black women deserve the freedom to self-determine if, when, and how they become parents. Reproductive justice moves beyond advocacy for reproductive rights, or the protections for access to reproductive health services under the law (e.g., sex education, contraceptive care, abortion care), to advocating for a moral and socially just approach to broadly promoting sexual and reproductive freedoms, particularly among historically marginalized communities.

SisterSong Women of Color Reproductive Justice Collective, founded in 1997 by several of the original founders of the reproductive justice framework, is the leading reproductive justice coalition in the U.S. today [19]. Although reproductive justice was a tool created by Black women and has been used by Black women to advocate and organize around the sexual and reproductive liberation of Black women, the framework has expanded to include any population that experiences reproductive oppression. Reproductive justice advocates for everyone to have the right to [19]:

- have a child
- not have a child
- maintain personal bodily autonomy
- parent their children in safe and sustainable environments

The reproductive justice framework, in its human rights–based approach, takes a holistic view of individuals and sees the interconnections between sexual health, reproductive health, and overall health. Although the title of the framework does not explicitly name sexual health, the founders highlight the need for people to have control over their sexual lives and sexual health [6]. Employing a sexual and reproductive justice perspective is essential to understanding and helping to mitigate the role intersecting structural, sexual, and reproductive oppressions have had on the lives of women and girls across the globe, including inequities in HIV diagnoses, treatment, and prevention [20, 21••, 22].

Reproductive justice advocates and organizations, like SisterLove, Inc. [23], support the integration of comprehensive sexual and reproductive health care to address multiple components of wellness simultaneously [3]. Reproductive justice focuses on addressing structural barriers to optimal health and provides a platform to move away from focusing on preventing poor health outcomes to cultivating opportunities for reproductive health and well-being. The inclusion of positive frames regarding sexual and reproductive health can empower people to leverage available resources and acquire additional resources to support their health. This frame also offers guiding principles to institute policies and measures to effectively eliminate health inequities, such as supporting increasing access and reducing barriers to prevention technologies.

## Applying a Reproductive Justice Lens to LARC and HIV Prevention Technology Provision

**Fig. 1:**
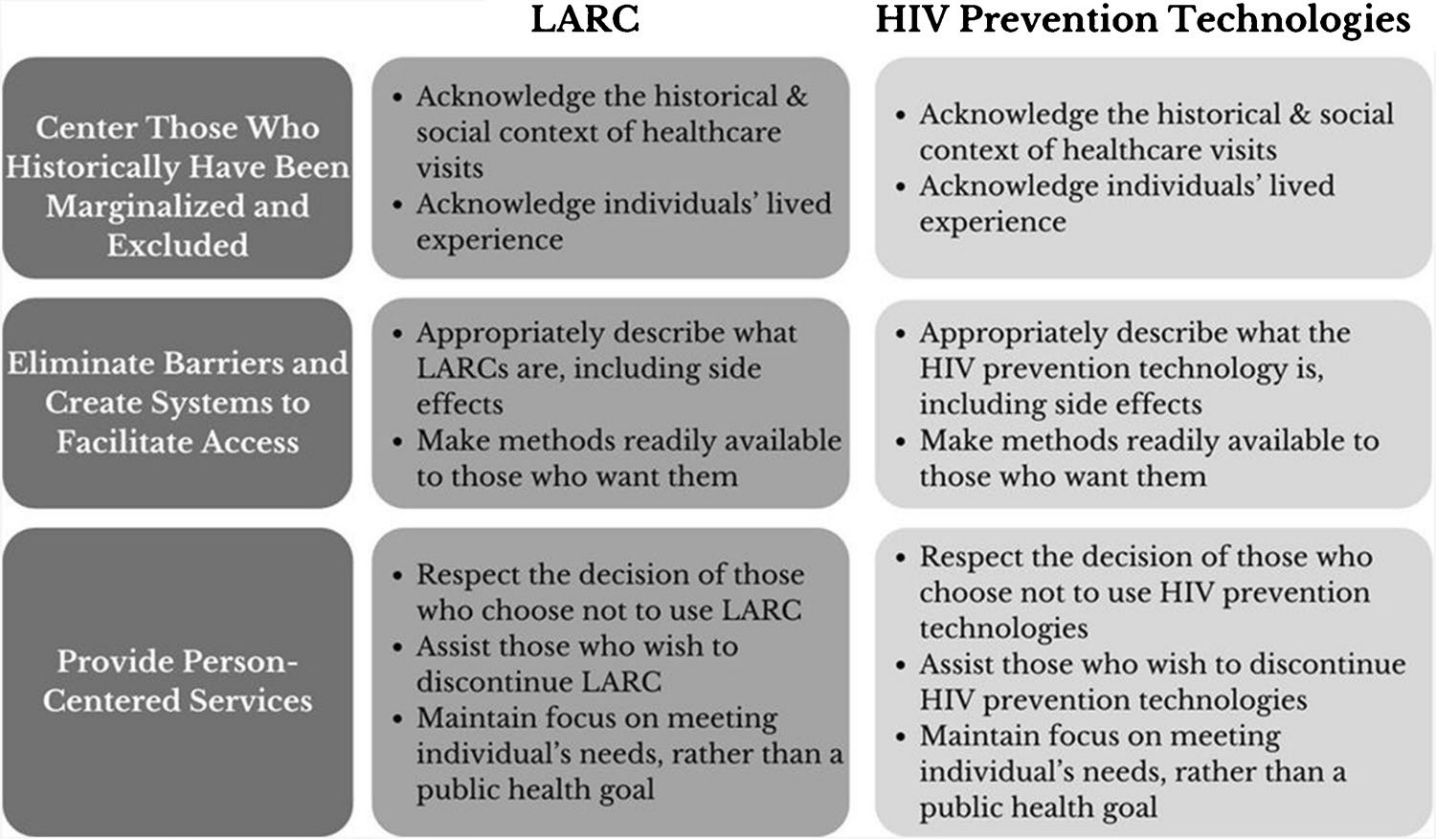
Applying principles of reproductive justice to sexual and reproductive health technologies

Reproductive health scholars have applied a reproductive justice lens to LARC implementation [6, 24]; here, we highlight some of the key principles of reproductive justice and apply them to emerging HIV prevention technologies (Fig. 1). Reproductive justice emphasizes the importance of centering and engaging with those who historically have been marginalized and excluded; eliminating barriers to access health-promoting technologies and creating systems to facilitate access; and employing person-centered approaches in the implementation of all health services work.

### Center Those Who Historically Have Been Marginalized and Excluded

A reproductive justice–based approach to implementing health technologies implores researchers and clinicians to value the knowledge, expertise, and advice of laypersons, advocates, and community members regarding how best to meet their needs [23]. As HIV continues to severely impact groups with social and personal identities that are stigmatized and marginalized, we must engage these groups in efforts to improve the science and implementation of HIV prevention tools. This engagement includes acknowledging the historical and social context for a health care visit and individuals’ lived experiences. As an example, this may include health care providers’ recognition that questions about sexual practices in the context of offering HIV prevention may be experienced as judgmental or discriminatory. In research, the use of participatory methods creates space to learn from people’s lived experiences [25], including those managing their sexual and reproductive health, navigating health care settings, and engaging with partners, families, and their community. This approach may also reveal structural and systemic barriers that must be addressed to facilitate equitable and person-centered utilization of prevention methods. Participatory research takes substantial time and funding, which are traditionally not supported by many funding agencies; advocating for equitable access to prevention technologies also includes advocating for marginalized groups to be involved in research design, development, and implementation.

### Eliminate Barriers and Create Systems to Facilitate Access to Prevention Technologies

Ensuring successful implementation of new prevention technologies requires expanded access to methods [2]. Potential users must have multiple accessible avenues for acquiring prevention information, services, and products. Although approaches to enhance access were emerging before the COVID-19 pandemic, the pandemic provided new opportunities as well as challenges. For example, the COVID-19 pandemic accelerated opportunities for telehealth, extended prescriptions, and utilization of self-administered prevention options by decades [26, 27••, 28, 29]. The expedited approval of self-administered subcutaneous medroxyprogesterone acetate in many locations is one example of expanding method mix and access. Clinics also creatively developed opportunities for remote counseling, followed by drive-by visits for injections, or access to medicines by mail or pharmacy. Despite the fact that these new opportunities expanded access for many, reliance on telehealth, or any one access pathway, also risks excluding certain groups. As new access approaches continue to emerge, it will be critical to evaluate for emerging inequities, and respond to those inequities with an intensification of outreach and support to meet specific communities’ needs better.

With the approval of multiple new PrEP products on the near horizon, the focus of reproductive justice on making methods available to those who need and want them becomes a clear opportunity and challenge. How to reconcile questions of cost, prioritization of specific populations, and limited (or lack of) data in other populations (pregnant and lactating people, for example) remains to be determined. Moreover, as multipurpose technologies become available, ensuring that people can seamlessly start a method that prevents pregnancy and HIV, decide to get pregnant and continue their HIV prevention without the contraceptive component, and then return to a multipurpose method postpartum will be critical to meeting people wherever they are in their reproductive and sexual health trajectories. Beyond method availability, how to effectively communicate about each method and share information about side effects and risks with diverse communities requires community engagement. Moreover, conveying nuanced messaging on method safety and that methods are novel and, therefore, additional safety or risk data may emerge requires ongoing collaboration with communities, advocates, and allies.

### Provide Person-Centered Services

Person-centered care emphasizes meeting an individual’s sexual and reproductive preferences, needs, and goals, irrespective of public health targets. With LARC and HIV prevention, this includes using person-centered counseling approaches, such as shared decision-making. In a counseling visit utilizing shared decision-making, the patient is recognized as the expert regarding their lived experience, life situations, needs, and preferences for sexual and reproductive health prevention and care [30, 31••]. The conclusion of such a counseling conversation may include patients choosing not to use a prevention method or discontinuation of a more effective method for a less effective method (or no method at all). This is in sharp contrast to tiered effectiveness or other forms of directive counseling, such as LARC first approaches. Particularly for longer acting forms of prevention that require provider participation in discontinuation, and also to facilitate patients’ communication about discontinuation of any type of method, it is critical for care to be offered in a manner free of shame or judgement. Acknowledging the history of contraceptive coercion, it may behoove clinicians and counselors to discuss access to removal or discontinuation services at the time of method initiation, signaling to patients the importance of person-centered services.

Even for providers and counselors who strive to provide person-centered services, their work is influenced by clinic- and system-level goals and metrics. When these goals and metrics include targets such as the number of LARCs placed, or the number of PrEP starts, core values of person-centered care can be deemphasized. On the one hand, clinic-level data on specific method initiation demonstrates access; if no LARCs or PrEP are initiated, there are likely access barriers that need to be addressed. To facilitate identification of access barriers while also supporting person-centered services, contraceptive researchers have suggested the use of a “floor,” rather than a target, for prevention methods [32]. These then can be coupled with person-centered measures of care experiences and other measures of sexual and reproductive well-being [33••]. Throughout the process of measure development, reproductive justice principles, such as centering voices that are most marginalized, may facilitate new ways to define success, and in turn promote the sexual and reproductive well-being of a broader group of people.

## Conclusion

Long-acting methods in contraception, HIV prevention, and multipurpose prevention provide an essential option for people to manage and achieve their sexual and reproductive health goals. Lessons learned from LARC suggest the importance of attention to the historical and ongoing sociopolitical context from the start—centering the voices of those often excluded in research, implementation, and evaluation. While HIV prevention and pregnancy prevention are certainly different, making the same mistakes in PrEP rollout as were demonstrated in LARC implementation is unacceptable. To mitigate potential missteps in HIV prevention service provision, clinicians, researchers, advocates, and policymakers can learn from the sexual and reproductive justice framework to develop, research, and implement new products. Additionally, a holistic and integrated sexual and reproductive health approach can support the continued breaking down of siloed reproductive and sexual health care systems. The ultimate goal of applying a reproductive justice lens to sexual and reproductive health is to support individuals’ ability to exercise sexual and reproductive autonomy, and address the social and structural determinants that perpetuate inequities in HIV and other sexual and reproductive health outcomes.

## References

[1] Crankshaw TL, Smit JA, Beksinska ME (2016). Placing contraception at the centre of the HIV prevention agenda. Afr J AIDS Res.

[2] Delany-Moretlwe S, Mullick S, Eakle R, Rees H (2016). Planning for HIV preexposure prophylaxis introduction: lessons learned from contraception. Curr Opin HIV AIDS.

[3] Seidman D, Weber S, Carlson K, Witt J (2018). Family planning providers’ role in offering PrEP to women. Contraception.

[4] Roberts D. Killing the black body: race, reproduction, and the meaning of liberty. Vintage; 2014.

[5] Perritt J (2020). #WhiteCoatsForBlackLives—addressing physicians’ complicity in criminalizing communities. N Engl J Med.

[6] Ross L, Solinger R. Reproductive justice: an introduction. Univ of California Press; 2017.

[7] Ott MA, Sucato GS (2014). Contraception for adolescents. J Pediatr.

[8] McNicholas C, Peipert JF (2012). Long-acting reversible contraception for adolescents. Curr Opin Obstet Gynecol.

[9] American College of Obstetricians and Gynecologists (2009). ACOG Committee Opinion no. 450: Increasing use of contraceptive implants and intrauterine devices to reduce unintended pregnancy. Long-Acting Reversible Contraception Working Group. Obstet and Gynecol.

[10] Brandi K, Fuentes L (2020). The history of tiered-effectiveness contraceptive counseling and the importance of patient-centered family planning care. Am J Obstet Gynecol.

[11] Jenkins A. Contraceptive coercion, access, and sex education. SIECUS. Available from: https://siecus.org/contraceptive-coercion-access-and-sex-education/.

[12] Miller E, Jordan B, Levenson R, Silverman JG (2010). Reproductive coercion: connecting the dots between partner violence and unintended pregnancy. Contraception.

[13] Wang F, Gu B, Cai Y. The end of China’s one-child policy. 2016. Available from: https://www.brookings.edu/articles/the-end-of-chinas-one-child-policy/.

[14] Spain JE, Peipert JF, Madden T, Allsworth JE, Secura GM (2010). The Contraceptive CHOICE Project: recruiting women at highest risk for unintended pregnancy and sexually transmitted infection. J Women’s Health.

[15] Gomez AM, Wapman M (2017). Under (implicit) pressure: young Black and Latina women’s perceptions of contraceptive care. Contraception.

[16] Amico JR, Bennett AH, Karasz A, Gold M (2016). She just told me to leave it: women’s experiences discussing early elective IUD removal. Contraception.

[17] Dehlendorf C, Levy K, Kelley A, Grumbach K, Steinauer J (2013). Women’s preferences for contraceptive counseling and decision making. Contraception.

[18] The United Nations. Universal Declaration of Human Rights. 1948.

[19] SisterSong. Reproductive justice. Available from: https://www.sistersong.net/reproductive-justice.

[20] UNAIDS. Women and HIV: a spotlight on adolescent girls and young women. 2019. Available from: https://www.unaids.org/en/resources/documents/2019/women-and-hiv.

[21] Ninsiima AB, Michielsen K, Kemigisha E, Nyakato VN, Leye E, Coene G (2020). Poverty, gender and reproductive justice. A qualitative study among adolescent girls in Western Uganda. Cult Health Sex.

[22] Maphalala N. Reproductive justice as human rights in the east and horn of Africa. In: Kioko C, Kagumire R, Matandela M, editors. Challenging patriarchy: the role of patriarchy. 2020. p. 119. Available from: https://ke.boell.org/sites/default/files/2020-05/Finalcopy-ChallengingPatriarchy.pdf.

[23] Ross L, Derkas E, Peoples W, Roberts L, Bridgewater P (2017). Radical reproductive justice: foundation, theory, practice, critique.

[24] Gubrium AC, Mann ES, Borrero S, Dehlendorf C, Fields J, Geronimus AT (2016). Realizing reproductive health equity needs more than long-acting reversible contraception (LARC). Am J Public Health.

[25] Keikelame MJ, Swartz L (2019). Decolonising research methodologies: lessons from a qualitative research project, Cape Town, South Africa. Glob Health Action.

[26] Touger R, Wood BR (2019). A review of telehealth innovations for HIV pre-exposure prophylaxis (PrEP). Curr HIV/AIDS Rep.

[27] Hoagland B, Torres TS, Bezerra DRB, Geraldo K, Pimenta C, Veloso VG (2020). Telemedicine as a tool for PrEP delivery during the COVID-19 pandemic in a large HIV prevention service in Rio de Janeiro-Brazil. Braz J Infect Dis.

[28] Nanda K, Lebetkin E, Steiner MJ, Yacobson I, Dorflinger LJ (2020). Contraception in the era of COVID-19. Glob Heal Sci Pract.

[29] Bateson DJ, Lohr PA, Norman WV, Moreau C, Gemzell-Danielsson K, Blumenthal PD (2020). The impact of COVID-19 on contraception and abortion care policy and practice: experiences from selected countries. BMJ Sex Reprod Health.

[30] Dehlendorf C, Krajewski C, Borrero S (2014). Contraceptive counseling: best practices to ensure quality communication and enable effective contraceptive use. Clin Obstet Gynecol.

[31] •• Sewell WC, Solleveld P, Seidman D, Dehlendorf C, Marcus JL, Krakower DS. Patient-led decision-making for HIV preexposure prophylaxis. Curr HIV/AIDS Rep. 2021;1–9. 10.1007/s11904-020-00535-w. **This article discusses new modes of offering people PrEP that include shared decision-making, in particular patient-led decision-making, to demonstrate respect for people’s reproductive autonomy and address provider-level barriers to HIV prevention method use.**

[32] Dehlendorf C, Bellanca H, Policar M (2015). Performance measures for contraceptive care: what are we actually trying to measure?. Contraception.

[33] Dehlendorf C, Akers AY, Borrero S, Callegari LS, Cadena D, Gomez AM (2021). Evolving the preconception health framework: a call for reproductive and sexual health equity. Obstet Gynecol.

